# Narrative Matters: Young people, social media and body image

**DOI:** 10.1111/camh.12345

**Published:** 2019-08-19

**Authors:** Victoria Goodyear

**Affiliations:** ^1^ School of Sport, Exercise and Rehabilitation Sciences University of Birmingham Birmingham UK

## Contemporary young people in a digital age

Social media is a ubiquitous element of young people's lives. It was recently reported that for 12‐ to 15‐year‐olds in the UK, 83% have their own smartphone, 99% go online for over 20 hours per week, and 69% have a social media profile (Ofcom, 2018). Many young people do not experience the online/offline binary that characterises the lives of older generations. Social media is a key resource in young people's lives for the development of identities and relationships, as well as emotional regulation, self‐expression, learning and much more (Third, Bellerose, Oliveira, Lala, & Theakstone, 2017). Disconnecting from social media is likely to be a challenge and unthinkable for many contemporary young people.

The digital lives of contemporary young people greatly differ from many adults. This has inevitably created challenges for many teachers, parents and clinicians who have a responsibility for young people's health and well‐being. There is already evidence that many adults feel helpless as they watch youth engaging with the tsunami of digital content and, as a result, they are tending to cede control to technology and social media companies (Goodyear & Armour, 2019). All of this suggests that teachers, parents and clinicians require access to the latest evidence‐based guidance to help protect young people from risk and to embrace the opportunities on offer.

## Social media and body image

One aspect of social media uses that adults frequently report to be concerning is in relation to body image. Extending the well‐established and profound influence of passive media (e.g. TV, magazines), social media is a mass user‐generated space in which young people can access and coproduce videos and images related to the body. Indeed, the rise of ‘healthism’ (an ideological, neoliberal and public construct of health) and concerns about individual autonomy, self‐monitoring and obsession/addiction from social media seen in adults are concerns in youth that are growing (Burnette, Kwitowski, & Mazzeo, 2017).

In order to offer support that will be effective, it is important for adults to be aware of the risks as well as appreciative of the potential benefits of social media use for young people's health and well‐being, particularly in relation to body image. For example, there is a growing body of evidence that suggests social media can be a positive educational health and well‐being resource (Goodyear & Armour, 2019). Research has highlighted that social media provides a context for young people to access social and emotional support, as well as develop a critical awareness of a diverse range of information and content (Third et al., 2017).

## Young people as users and generators

Social media is a highly interactive space inhabited by young people. In order to understand the relationship between social media use and body image, it is therefore important to not only focus on the information that is accessible to young people, but also the content that young people interact with, mobilise and generate.

In relation to body image, it is well established that self‐presentation on social media is of central importance to young people, and can drive the ways in which young people participate, interact and communicate (Handyside & Ringrose, 2017). Across a range of different mediums, it is certainly evident that many young people post photographs of their bodies in ways that conform to particular body ideals, such as through the use of selfies and/or filters (Burnette et al., 2017). This behaviour can have an affective/emotive influence on how other young people their age feel they should look, extending, for example, the traditional influence of celebrities from magazines (Goodyear & Armour, 2019). In addition, the images that young people share of themselves and their bodies while, perhaps, posted transiently, can take on an unanticipated life. The self‐focused behaviour can invite personal judgement, ridicule and criticism. ‘Teen’ produced content can also circulate widely and be used within peer networks as a form of digital currency. In turn, young people can become obsessed with the ways they look on social media, and it has been suggested that some are addicted to the feedback they obtain on whether their bodies conform to socially acceptable standards (Handyside & Ringrose, 2017).

## A focus on content

To help adults better understand the ways in which social media shapes and influences young people's health and well‐being, a focus on the ways in which young people use and generate content can be helpful. Informed by a pedagogical perspective, content is not only information, such as images related to particular types of bodies (Goodyear & Armour, 2019). Content in the context of the user‐generated spaces of social media is more dynamic and involves the interactive functionalities (likes, algorithms) in the construction of content, as well as how content is mobilised, and reaches and influences users. In recent research coproduced with young people, five forms of social media content were reported to be influential on health‐related knowledge and behaviours (Goodyear, Armour, & Wood, 2018; see Table 1). These forms of content include a dual focus on the accessibility of information, and the ways in which young people generate content. One approach for relevant adults, therefore, is to use these forms of content to understand and engage with young people's needs and behaviours, and/or to design programmes and interventions to ensure that they are relevant and authentic to young people's needs and interests (see Goodyear et al., 2018). For example, automatically sourced content can be used to engage young people in a critical analysis of how they have come to understand different bodies, and what they conceptualise as a ‘healthy body’. Alternatively, the role of likes can be used in interventions to help young people critically evaluate moral and ethical behaviours in conceptualisations of information coproduced on social media about body image.

**Table 1:** Different types of social media content that influenced young people's health‐related knowledge and behaviours in different ways (Goodyear et al., 2018, p. 19)

Content	Explanation
Automatically Sourced Content	The influence of health‐related material that social media sites preselect and promote to young people. For example, Instagram preselects content that users see on the ‘search and explore’ feature, based on a user's likes, who that user follows and their followers’ likes, and automatically sourced accounts
Suggested Content	The process whereby young people's ‘searches’ for specific health‐related material result in social media sites then promoting vast amount of partially related material to their accounts. For example, suggested videos on YouTube
Peer Content	Content created and shared by peers, and the actions of young people liking or not liking posts, had a powerful influence over young people's health‐related behaviours and understandings. Young people experienced a level of peer pressure to change their behaviours as a result of viewing health‐related material shared by peers, including selfies. Young people developed shared understandings about health from sharing and creating content in health‐related spaces
Likes	Likes are positioned as a form of endorsement and had a strong influence on young people's engagement with health‐related material and their health‐related understandings and behaviours. Credibility of information is gauged by the number of likes a post receives, with 200 likes acting as the benchmark
Reputable Content	The influences of specific social media accounts on young people's health‐related understandings and behaviours. These types of accounts have a high number of followers and this provides a powerful platform from which to reach and impact young people in both positive and negative ways. Celebrities acted as role models, yet their posts and/or advertisements were often inappropriate and/or targeted at adult health‐related behaviours

## Actions and advice for adults

There is clear evidence that social media is a very powerful educative health resource that has considerable significance in the lives of young people. Most young people experience positive impacts and are critically aware users and generators of social media. While the health‐related risks of social media should not be excluded, adults must focus on supporting young people to engage with social media so that they can realise more of the positive impacts on their health and well‐being, particularly in the areas of body image. The health‐related risks of social media should not be ignored, but an action for adults is to become suitably digital literate so that they can promote positive outcomes and offer support to young people at times of vulnerability. A focus on digital literacy for adults would see adults able to critically evaluate different forms of content for their own and young people's lives, as well as developing the necessary digital skills so that they can navigate digital mediums and offer appropriate support.

To help adults develop a better awareness of how the forms of content impact on young people, evidence‐based training resources can be accessed from: http://opencpd.net/Guidelines.html.

## Ethical information

No ethical approval was required for this article.
